# Magnesium in Prevention and Therapy

**DOI:** 10.3390/nu7095388

**Published:** 2015-09-23

**Authors:** Uwe Gröber, Joachim Schmidt, Klaus Kisters

**Affiliations:** 1 Academy of Micronutrient Medicine, Essen 45130, Germany; E-Mails: Prof.schmidt.dd@t-online.de (J.S.); kisters@annahospital.de (K.K.); 2 Department of Internal Medicine I, St. Anna-Hospital, Herne 44649, Germany

**Keywords:** magnesium, hypomagnesemia, cardiovascular disease, diabetes mellitus, asthma, ADHD, Alzheimer’s disease

## Abstract

Magnesium is the fourth most abundant mineral in the body. It has been recognized as a cofactor for more than 300 enzymatic reactions, where it is crucial for adenosine triphosphate (ATP) metabolism. Magnesium is required for DNA and RNA synthesis, reproduction, and protein synthesis. Moreover, magnesium is essential for the regulation of muscular contraction, blood pressure, insulin metabolism, cardiac excitability, vasomotor tone, nerve transmission and neuromuscular conduction. Imbalances in magnesium status—primarily hypomagnesemia as it is seen more common than hypermagnesemia—might result in unwanted neuromuscular, cardiac or nervous disorders. Based on magnesium’s many functions within the human body, it plays an important role in prevention and treatment of many diseases. Low levels of magnesium have been associated with a number of chronic diseases, such as Alzheimer’s disease, insulin resistance and type-2 diabetes mellitus, hypertension, cardiovascular disease (e.g., stroke), migraine headaches, and attention deficit hyperactivity disorder (ADHD).

## 1. Introduction

Magnesium is the eight most common element in the crust of the Earth and is mainly tied up within mineral deposits, for example as magnesite (magnesium carbonate) and dolomite. Dolomite CaMg (SO_3_)_2_ is as the name suggests abundant in the Dolomite mountain range of the Alps [1,2,3]. The most plentiful source of biologically available magnesium, however, is the hydrosphere (*i.e.*, oceans and rivers). In the sea, the concentration of magnesium is about 55 mmol/L and in the Dead Sea as an extreme example, the concentration is reported to be 198 mmol/L magnesium and has steadily increased over time [4]. Magnesium is an essential electrolyte for living organisms and is the fourth most abundant mineral in the human body. Humans need to consume magnesium regularly to prevent magnesium deficiency, but as the recommended daily allowance for magnesium varies, it is difficult to define accurately what the exact optimal intake should be. Based on magnesium’s many functions within the human body, it plays an important role in prevention and treatment of many diseases. Low levels of magnesium have been associated with a number of chronic and inflammatory diseases, such as Alzheimer’s disease, asthma, attention deficit hyperactivity disorder (ADHD), insulin resistance, type-2 diabetes mellitus, hypertension, cardiovascular disease (e.g., stroke), migraine headaches, and osteoporosis [5].

## 2. Functions of Magnesium

Magnesium is primarily found within the cell where it acts as a counter ion for the energy-rich ATP and nuclear acids. Magnesium is a cofactor in more than 300 enzyme systems that regulate diverse biochemical reactions in the body, including protein synthesis, muscle and nerve transmission, neuromuscular conduction, signal transduction, blood glucose control, and blood pressure regulation. Some magnesium dependent enzymes are Na^+^/K^+^-ATPase, hexokinase, creatine kinase, protein kinase, and cyclases (see Table 1). Magnesium is also necessary for structural function of proteins, nucleic acids or mitochondria. It is required for DNA and RNA synthesis, and for both aerobic and anaerobic energy production—oxidative phosphorylation and glycolysis—either indirectly as a part of magnesium-ATP complex, or directly as an enzyme activator.

Magnesium also plays a key role in the active transport of calcium and potassium ions across cell membranes, a process that is important for nerve impulse conduction, muscle contraction, vasomotor tone and normal heart rhythm. As natural calcium antagonist the block of *N*-methyl-d-aspartate (NMDA) receptor channels by external magnesium is believed to be of great physiological importance. Moreover, it contributes to the structural development of bone and is required for the adenosine triphosphate-dependent synthesis of the most important intracellular antioxidant glutathione [6,7,8,9,10,11].

The most important reservoir for magnesium is the bone (about 60% of total body magnesium), the remaining 40% is located extra- and intracellularly. Magnesium excretion is mainly regulated by the kidney. About 100 mmol/L magnesium is filtered daily [12,13,14,15]. The total magnesium content of the human body is reported to be ~20 mmol/kg of fat-free tissue. In other words, total magnesium in the average 70 kg adult with 20% (w/w) fat is ~1000 to 1120 mmol or ~24 g [10,13,15].

Magnesium is beside sodium, potassium and calcium an important electrolyte for human metabolism. About 99% of total body magnesium is located in bone, muscles and non-muscular soft tissue [12,13]. Approximately 50%–60% of magnesium resides as surface substituents of the hydroxyapatite mineral component of bone. Most of the remaining magnesium is contained in skeletal muscle and soft tissue. The magnesium content of bone decreases with age, and magnesium that is stored in this way is not completely bioavailable during magnesium deprivation.

**Table 1:** Functions of magnesium (selection) [6,7,8,9,10].

Magnesium is involved in more than 300 essential metabolic reactions (e.g., all Adenosine Triphosphate (ATP)-dependent reactions).
**Energy production** (→ ATP production)
Breakdown and energetic utilization of carbohydrates, proteins and fats in intermediate metabolism (e.g., glycolysis, respiratory chain phosphorylation). ATP exists primarily as a complex with magnesium (MgATP).
**Enzyme activation** (examples)
Mitochondrial ATP synthase, Na^+^/K^+^-ATPase, Hexokinase, Creatine kinase, Adenylate cyclase, Phosphofructokinase, tyrosine kinase activity of the insulin receptor.
**Calcium antagonist/NMDA-receptor antagonist**
Control of calcium influx at the cell membrane (course of contractions, regulation of vascular muscle tone): muscle contraction/relaxation, neurotransmitter release, action potential conduction in nodal tissue, neuromuscular impulse conduction (inhibition of calcium-dependent acetylcholine release at the motor end plate), maintenance and stabilization of membrane physiology, muscle contraction.
**Cardiovascular system**
Economization of cardiac pump function, regulation of potassium movement in myocardial cells, protection against stress, vasodilation of the coronary and peripheral arteries, reduction of platelet aggregation.
**Membrane function**
Transmembrane electrolyte flux, active transport of potassium and calcium across cell membranes, regulation of cell adhesion and cell migration.
**Structural roles**
Component of mineralized bone (structure, microarchitecture), multiple enzyme complexes, mitochondria, proteins, polyribosomes, and nucleic acids.
**Nutrient metabolism**
Metabolic activation and utilisation of vitamin D, B-vitamins (e.g., thiamine) and glutathione.

Intracellular magnesium concentrations range from 5–20 mmol/L; 1%–5% is ionized, the remainder is bound to proteins, negatively charged molecules and adenosine triphosphate (ATP) [14,15]. Extracellular magnesium accounts for about 1%–3% of total body magnesium [13,15] which is primarily found in serum and red blood cells. Normal serum magnesium concentration is about 0.76–1.15 mmol/L [7,16,17,18,19]. It is categorized into three fractions. It is either ionized (55%–70%), bound to protein (20%–30%) or complexed with anions (5%–15%) such as phosphate, bicarbonate and citrate or sulphate. Red blood cells/serum magnesium ratio is about 2.8 [14,15].

## 3. Magnesium and Nutrition

Dietary surveys of people in Europe and in the United States still reveal that intakes of magnesium are lower than the recommended amounts [20,21,22]. Epidemiological studies in Europe and North America have shown that people consuming Western-type diets are low in magnesium content, *i.e*. <30%–50% of the RDA for magnesium. It is suggested that the dietary intakes of magnesium in the United States have been declining over the last 100 years from about 500 mg/day to 175–225 mg/day. This is likely a result of the increasing use of fertilizers and processed foods [5,9,22,23,24]. In 1997, the Food and Nutrition Board (FNB) of the Institute of Medicine had increased the dietary references intakes (RDA) for magnesium, based on the results of controlled balance studies. The new RDA ranges from 80 mg/day for children 1–3 year of age to 130 mg/day for children 4–8 year of age. For older males, the RDA for magnesium ranges from as low as 240 mg/day (range, 9–13 year of age) and increases to 420 mg/day for males 31–70 year of age and older. For females, the RDA for magnesium ranges from 240 mg/day (9–13 year of age) to 360 mg/day for females 14–18 year of age. The RDA for females 31–70 year of age and older is 320 mg/day [6].

Water accounts for ~10% of daily magnesium intake [25], chlorophyll (and thus green vegetables such as spinach) is the major source of magnesium. Nuts, seeds and unprocessed cereals are also rich in magnesium. Legumes, fruit, fish and meat have an intermediate magnesium concentration. Some types of food processing, such as refining grains in ways that remove the nutrient-rich germ and bran, lower magnesium content substantially. Low magnesium concentrations are found in dairy products, except milk [24,26].

The United States NHANES 2005–2006 survey reported that nearly one half of all American adults have an inadequate intake from food and water of magnesium and do not consume the estimated average requirements (EAR) (set at 255–350 mg depending on gender and age group) [27,28]. A chronic magnesium deficiency (serum magnesium <0.75 mmol/L) is associated with an increased risk of numerous preclinical and clinical outcomes, including atherosclerosis, hypertension, cardiac arrhythmias, stroke, alterations in lipid metabolism, insulin resistance, metabolic syndrome, type 2 diabetes mellitus, osteoporosis as well as depression and other neuropsychiatric disorders. Furthermore, magnesium deficiency may be at least one of the pathophysiological links that may help to explain the interactions between inflammation and oxidative stress with the aging process and many age-related diseases [5,7,11,22,27,29,30,31,32,33,34].

## 4. Magnesium Absorption and Excretion

**Figure 1:**
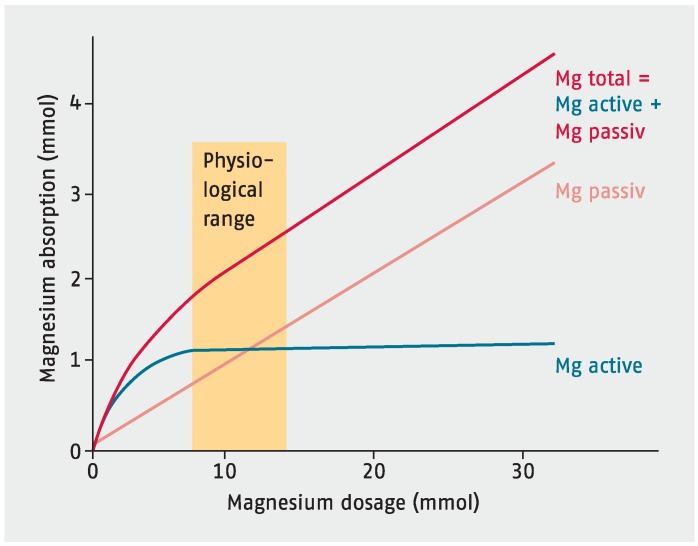
Magnesium absorption.

Magnesium homeostasis is maintained by the intestine, the bone and the kidneys. Magnesium is mainly absorbed in the small intestine, which was shown by ^28^Mg isotope measurements, although some is also taken up via the large intestine [15,35]. Of the total dietary magnesium consumed, only about 24%–76% is absorbed in the gut the rest is eliminated in the faeces [15,36]. The majority of magnesium is absorbed in the small intestine by a passive paracellular mechanism, which is driven by an electrochemical gradient and solvent drag (see Figure 1). Paracellular magnesium absorption is responsible for 80%–90% of intestinal magnesium uptake. The driving force behind this passive magnesium transport is supplied by the high luminal magnesium concentration, which ranges between 1.0 and 5.0 mmol/L, and the lumen-positive transepithelial voltage of ~15 mV [37]. Paracellular magnesium absorption relies on tight junction permeability, which is still poorly understood. The ileum and distal parts of the jejunum are known to be the most permeable for ions because of the relatively low expression of “tightening” claudins 1, 3, 4, 5 and 8 [37,38,39]. As such, paracellular magnesium transport seems mainly restricted to these areas that lack the “tightening” claudins. The exact mechanism facilitating paracellular magnesium absorption still remains unknown. A minor, yet important, regulatory fraction of magnesium is transported via the transcellular transporter transient receptor potential channel melastatin member TRPM 6 and TRPM 7—members of the long transient receptor potential channel family—which also play an important role in intestinal calcium absorption [40,41].

It is worth noting that intestinal absorption is not directly proportional to magnesium intake but is dependent mainly on magnesium status. The lower the magnesium level, the more of the mineral is absorbed in the gut, thus relative magnesium absorption is high when intake is low and vice versa. The kidneys are crucial in magnesium homeostasis as serum magnesium concentration is primarily controlled by its excretion in urine. Under physiological conditions, ~2400 mg of magnesium in plasma is filtered by the glomeruli. Of the filtered load, ~2300 mg is immediately reabsorbed and only 3%–5% is excreted in the urine, *i.e.*, ~100 mg [36]. Only little magnesium is reabsorbed in the proximal tubule. Most of the filtered magnesium is reabsorbed in the loop of Henle, mostly in the thick ascending limb (up to 70% of total magnesium reabsorption). The reabsorption and excretion of magnesium is influenced by several not yet classified mechanisms. In this context, we could show that an overload of blood cells with magnesium in renal insufficiency can be avoided by a special cell membrane buffering system for magnesium. In severe forms of renal insufficiency, this buffering system for magnesium is destroyed and an overload with magnesium in human cells is observed [42]. Furthermore, the exchange time for magnesium between intra- and extracellular pools is relatively long [12,13]. Hypomagnesaemia is frequently linked with hypokalemia owing to disturbances in renal secretion of potassium in the connecting tubule and collecting duct [12,37].

Magnesium absorption and excretion is influenced by different hormones. It has been shown that 1,25-dihydroxyvitamin D [1,25(OH)_2_D] can stimulate intestinal magnesium absorption. On the other hand, magnesium is a cofactor that is required for the binding of vitamin D to its transport protein, vitamin D binding protein (VDBP). Moreover, conversion of vitamin D by hepatic 25-hydroxlation and renal 1α-hydroxylation into the active, hormonal form 1,25(OH)_2_D is magnesium-dependent. Magnesium deficiency, which leads to reduced 1,25(OH)_2_D and impaired parathyroid hormone response, has been implicated in “magnesium-dependent vitamin-D-resistant rickets”. Magnesium supplementation substantially reversed the resistance to vitamin D treatment [43,44]. Next to 1,25(OH)_2_D, several other factors, such as oestrogen or parathyroid hormone (PTH), are involved in the magnesium excretion. Oestrogen is known to stimulate TRPM6 expression [45]. Thus, oestrogen substitution therapy can normalize hypermagnesuria, which occurs frequently in postmenopausal women. Interestingly, TRPM6 expression appears to be regulated by serum magnesium levels and oestrogens, but not by 1,25(OH)_2_D or PTH action [37].

Of special importance is PTH. Absorption of both magnesium and calcium appears to be inter-related, with concomitant deficiencies of both ions well described. For example, the stimulation of PTH secretion in response to hypocalcemia acts to restore the serum calcium concentration to normal. Hypomagnesemia impairs hypocalcemic-induced PTH release, which is corrected within in minutes after infusion of magnesium. The rapidity of correction of PTH concentrations suggests that the mechanism of action of magnesium is enhanced release of PTH. Magnesium is also required for the sensitivity of the target tissues to PTH. Calciotrophic hormones, such as PTH, have profound effects on magnesium homeostasis. PTH release enhances magnesium reabsorption in the kidney, absorption in the gut and release from the bone [37,46,47]. PTH influences magnesium absorption, however, hypercalcemia antagonizes this effect. In this context, different findings have often been described in primary hyperparathyroidism. Also in Addison’s disease as well as in spironolactone treated patients, magnesium excretion is slightly decreased [48,49].

In recent years, gene-linkage studies in families with hypomagnesemia have been performed. Some of these diseases are familial hypomagnesemia with hypercalciuria and nephrocalcinosis. Mutations in the claudin-16 gene have been shown to be responsible for this rare inherited disorder. Bartter’s syndrome is often linked to mild hypomagnesemia. It belongs to a group of autosomal-recessive disorders characterized by reduced salt absorption in the thick ascending limb. Mutations in the TRPM6 gene are associated with hypomagnesemia and secondary hypocalcemia. Other human magnesium genetic transport disorders are the isolated autosomal recessive hypomagnesemia (gene: Epidermal Growth Factor/EGF), autosomal dominant hypomagnesemia (gene: potassium channel, voltage gated shaker related subfamily A, member 1/KCNA1), Gitelman syndrome (gene: Na-Cl-Co-transporter/NCC), isolated dominant hypomagnesemia (gene: Na^+^/K^+^-ATPase), maturity-onset diabetes of young (gene: Hepatocyte Nuclear Factor-1 Beta/HNF1B), and the SeSAME syndrome (gene: potassium channel, inwardly rectifying subfamily J, member 10/KCNJ10) [15,37].

## 5. Magnesium Status

Assessing magnesium status is difficult because most magnesium is inside the cells or in bone [9,12]. The most common and valuable test in clinical medicine for the rapid assessment of changes in magnesium status is the serum magnesium concentration, even though serum levels have little correlation with total body magnesium levels or concentrations in specific tissues. Only 1% of total body magnesium is present in extracellular fluids, and only 0.3% of total body magnesium is found in serum [7,15,16,50,51]. In healthy individuals, magnesium serum concentration is closely maintained within the physiological range. The normal reference range for the magnesium in blood serum is 0.76–1.15 mmol/L [7,16,17,18,19].

According to many magnesium researchers, the appropriate lower reference limit of the serum magnesium concentration should be 0.85 mmol/L, especially for patients with diabetes [17,18,52,53]. For example, in the NHANES I study the reference interval for serum magnesium was determined in 15,820 individuals between the ages of 18 and 74 years. The results of this study identified the reference interval as 0.75 mmol/L to 0.955 mmol/L with a mean concentration of 0.85 mmol/L[54]. In a European study, magnesium deficiency was determined clinically and compared with the serum magnesium concentration. It was found that in individuals with serum magnesium level of 0.70 mmol/L, 90% of the individuals had clinical magnesium deficiency and at a cut off magnesium level of 0.75 mmol/L, 50% of individuals had clinical magnesium deficiency. At a cut off level of 0.80 mmol/L, 10% of individuals had clinical magnesium deficiency and at a cut off of 0.90 mmol/L, only 1% of the individuals had clinical magnesium deficiency [55]. A cohort of 9784 participants in the NHANES I study was followed for 18 years. There were 690 participants who developed type 2 diabetes mellitus. Using an adjusted Cox’s regression, the authors showed that the hazard ratio was 1.20 with a serum magnesium concentration between 0.80 and 0.84 mmol/L and the hazard ratio was 1.51 when the serum magnesium concentration was <0.80 mmol/L. The risk ratio began to increase when the serum magnesium level was <0.85 mmol/L [56]. After all, lower magnesium levels appear to be associated with a more rapid decline of renal function in patients with type 2 diabetes. Patients with serum magnesium levels between 0.82 and 1.03 mmol/L had the lowest deterioration of renal function and the best glycemic control [57,58].

The ionized magnesium concentration and the magnesium loading (or tolerance) test have been shown to be more accurate. The reference range for serum ionised magnesium concentration is 0.54–0.67 mmol/L [7,10,36]. In the magnesium loading test, the percentage of magnesium retained after parenteral administration of magnesium is determined. Up until today, no single method is considered satisfactory. Although some limitations may apply, serum magnesium concentration is still used as the standard for evaluating magnesium status in patients [15]. To comprehensively evaluate magnesium status, both laboratory tests and the clinical assessment of magnesium deficit symptoms might be required.

## 6. Magnesium Deficiency

Severe hypermagnesemia or magnesium intoxication appears very seldom in human disease. Such conditions only occur in severe renal insufficiency or iatrogen [13,42,59]. However, clinical symptoms are observed more frequently in magnesium deficient and insufficient patients in internal medicine. Magnesium deficiency is not uncommon among the general population: its intake has decreased over the years especially in the Western world. Hypomagnesaemia is defined as serum magnesium concentration <0.75 mmol/L. Early signs of magnesium deficiency are non-specific and include loss of appetite, lethargy, nausea, vomiting, fatigue, and weakness. More pronounced magnesium deficiency presents with symptoms of increased neuromuscular excitability such as tremor, carpopedal spasm, muscle cramps, tetany and generalized seizures. Hypomagnesemia can cause cardiac arrhythmias including atrial and ventricular tachycardia, prolonged QT interval and torsades de pointes (see also Table 2) [17,18,36,59,60,61,62].

Hypomagnesaemia is frequently associated with other electrolyte abnormalities such as hypokalemia and hypocalcaemia. Conditions that may lead to hypomagnesemia include alcoholism, poorly-controlled diabetes, malabsorption (e.g., Crohn’s disease, ulcerative colitis, coeliac disease, short bowel syndrome, Whipple’s disease), endocrine causes (e.g., aldosteronism, hyperparathyroidism, hyperthyroidism), renal disease (e.g., chronic renal failure, dialysis, Gitelman’s syndrome) and medication use. A variety of drugs including antibiotics, chemotherapeutic agents, diuretics and proton pump inhibitors can cause magnesium loss and hypomagnesemia (see Table 3). In addition, magnesium deficiency exacerbates potassium mediated arrhythmia, in particular in the presence of digoxin intoxication [63,64,65].

**Table 2:** Magnesium: Deficiency signs and symptoms [7].

**General**: Anxiety, lethargy, weakness, agitation, depression, dysmenorrhea, hyperactivity, headache, irritability, dysacusis, low stress tolerance, loss of appetite, nausea, sleep disorders, impaired athletic performance.
**Musculature**: Muscle spasm, cramps in the soles of the feet, leg cramps, facial muscles, masticatory muscles, and calves, carpopedal spasm, back aches, neck pain, urinary spasms, magnesium deficiency tetany.
**Nerves/CNS**: Nervousness, increased sensitivity of NMDA receptors to excitatory neurotransmitters, migraine, depression, nystagmus, paraesthesia, poor memory, seizures, tremor, vertigo.
**Gastrointestinal tract**: Constipation.
**Cardiovascular system**: Risk of arrhythmias, supraventricular or ventricular arrhythmias, hypertension, coronary spasm, decreased myocardial pump function, digitalis sensitivity, Torsade de pointes, death from heart disease.
**Electrolytes**: Hypokalaemia, hypocalcaemia, retention of sodium.
**Metabolism**: Dyslipoproteinemia (increased blood triglycerides and cholesterol), decreased glucose tolerance, insulin resistance, increased risk of metabolic syndrome, disturbances of bone and vitamin D metabolism, resistance to PTH, low circulating levels of PTH, resistance to vitamin D, low circulating levels of 25(OH)D, recurrence of calcium oxalate calculi.
**Miscellaneous**: Asthma, chronic fatigue syndrome, osteoporosis, hypertension, altered glucose homeostasis.
**Pregnancy**: Pregnancy complications (e.g., miscarriage, premature labor, eclampsia).

**Table 3:** Drug-induced magnesium loss and hypomagnesemia [63,64,65].

Drug Group (Drug Substance)	Mechanism/Effect
Aminoglycosides (e.g., gentamicin, tobramycin, amikacin)	increased renal magnesium loss, secondary hyperaldosteronism
Antimicrobial medication (Pentamidine)	increased renal magnesium loss
Antiviral medication (foscarnet)	nephrotoxicity, increased renal magnesium loss
Beta adrenergic agonists (e.g., Fenoterol, salbutamol, theophylline)	increased renal magnesium excretion, metabolic abnormalities (magnesium shift into cells)
Bisphosphonates (pamidronate)	renal impairment, magnesium excretion
Chemotherapeutic agents (e.g., amsacrine, cisplatin)	nephrotoxicity, cisplatin accumulates in renal cortex, increased renal magnesium loss
Immunosuppressants (cyclosporine, sirolimus)	2- to 3-fold increased urinary magnesium excretion (→ magnesium wasting)
Loop diuretics, esp. long-term use	increased renal magnesium loss, secondary hyperaldosteronism
(e.g., furosemide)	
Monoclonal antibody (e.g. cetuximab, panitumumab)	EGFR blockade in the nephron impairs the active transport of magnesium (→ magnesium wasting)
Polyene antifungals (amphotericin B)	nephrotoxicity
Proton pump inhibitors	loss of active magnesium absorption via transient receptor potential melastatin-6 and -7 (TRPM6/7)
Thiazide diuretics, esp. long-term use (e.g., hydrochlorothiazide)	increased renal magnesium loss, secondary hyperaldosteronism

## 7. Magnesium in the Treatment and Prevention of Diseases

Magnesium deficiency has been linked to atherosclerosis, alterations in blood lipids and blood sugar, type 2 diabetes, myocardial infarction, hypertension, kidney stones, premenstrual syndrome and psychiatric disorders [22,52,66,67,68,69,70]. A number of common clinical symptoms and diseases in association with magnesium deficiency are described in the following.

### 7.1. Magnesium, Type 2 Diabetes and Metabolic Syndrome

Diabetes mellitus, both type-1 and type-2, are among the most common causes of magnesium deficiency [34,71,72]. The incidence of hypomagnesemia in patients with type 2 diabetes ranges widely, from 13.5%–47.7% [34]. Causes include poor oral intake, increased renal loss and the chronic diarrhea associated with autonomic neuropathy. Drugs like proton-pump inhibitors can impair the gastrotintestinal absorption of magnesium. This effect may be the result of a drug-induced decrease in the pH of the intestinal lumen that alters the affinity of transient receptor potential melastatin-6 and melastastin-7 (TRPM6, TRPM7) channels on the apical surface of enterocytes for magnesium [34,73]. Probably one of the most studied chronic diseases with respect to magnesium is type 2 diabetes mellitus and the metabolic syndrome. Magnesium plays a crucial role in glucose and insulin metabolism, mainly through its impact on tyrosine kinase activity of the insulin receptor, by transferring the phosphate from ATP to protein. Magnesium may also affect phosphorylase b kinase activity by releasing glucose-1-phosphate from glycogen. In addition, magnesium may directly affect glucose transporter protein activity 4 (GLUT4), and help to regulate glucose translocation into the cell [5,67,71,72,74].

Recent studies have shown that magnesium intake is inversely associated with the incidence of type 2 diabetes. This finding suggests that increased consumption of magnesium-rich foods such as whole grains, beans, nuts, and green leafy vegetables may reduce the risk of diabetes type 2 [12,75,76]. A meta-analysis of seven prospective cohort studies from 1966–2007 investigated the association between magnesium intake (from foods only or from foods and supplements combined) and the incidence of type 2 diabetes. 286,668 participants and 10,912 cases were included. All but one study found an inverse relation between magnesium intake and risk of type 2 diabetes, and in four studies the association was statistically significant. The overall relative risk for a 100 mg magnesium intake per day was 0.85 (95% CI, 0.79–0.92). Results were similar for intake of dietary magnesium (RR, 0.86; 95% CI, 0.77–0.95) and total magnesium (RR, 0.83; 95% CI, 0.77–0.89) [76]. In a prospective study, the long-term associations of magnesium intake with incidence of diabetes, systemic inflammation, and insulin resistance among 4479 young American adults (age: 18–30 years old) were investigated [77]. Magnesium intake was inversely associated with incidence of diabetes after adjustment for potential confounders. The multivariable-adjusted hazard ratio of diabetes for participants in the highest quintile of magnesium intake was 0.53 (95% CI, 0.32–0.86; *p* < 0.01) compared with those in the lowest quintile. Consistently, magnesium intake was significantly inversely associated with high sensitivity CRP (hs-CRP), Interleukin 6 (IL-6), fibrinogen, and homeostasis model assessment as an index of insulin resistance (HOMA-IR), and serum magnesium levels were inversely correlated with hs-CRP and HOMA-IR [77].

Another recent meta-analysis of 13 prospective cohort studies involving 536,318 participants and 24,516 cases detected a significant inverse association between magnesium intake and risk of type 2 diabetes (relative risk (RR) 0.78 (95% CI 0.73–0.84)) [78]. A dietary intervention study examined the question if magnesium intake through food according to the Recommended Dietary Allowance (RDA) has an effect on insulin resistance among participants with metabolic syndrome. To examine the magnesium dose-response, and if the RDA (= 350 mg/day) was met, the magnesium intake was investigated with the outcome of HOMA-IR >3.6. Magnesium intake category variables were assessed over three time-points using linear mixed models. After adjustment for covariates, the likelihood of elevated HOMA-IR (>3.6) over time was 71% lower (OR: 0.29; 95% CI: 0.12, 0.72) in participants in the highest quartile of dietary daily magnesium intake (385.2 mg/day) compared to those in the lowest quartile (206.5 mg/day) at baseline [79]. A higher magnesium intake may be particularly beneficial in middle-aged persons among those with a high offsetting risk of metabolic impairment and developing diabetes. According to recent studies a higher magnesium intake may lower significant the risk of progressing from prediabetes to manifest diabetes [67,79]. In a double-blind placebo-controlled randomized trial a total of 116 men and non-pregnant women, aged 30–65 years with hypomagnesaemia and newly diagnosed with prediabetes, were enrolled to receive either 30 mL of MgCl_2_ 5% solution (equivalent to 382 mg of magnesium) or an inert placebo solution once daily for four months. The primary trial endpoint was the efficacy of magnesium supplementation in reducing plasma glucose levels. At the end of follow-up, fasting (86.9 ± 7.9 and 98.3 ± 4.6 mg/dL, respectively; *p* = 0.004) and post-load glucose (124.7 ± 33.4 and 136.7 ± 23.9 mg/dL, respectively; *p* = 0.03) levels, HOMA-IR indices (2.85 ± 1.0 and 4.1 ± 2.7, respectively; *p* = 0.04) and triglycerides (166.4 ± 90.6 and 227.0 ± 89.7, respectively; *p* = 0.009) were significantly decreased, whereas high density lipoprotein cholesterol (HDL) (45.6 ± 10.9 and 46.8 ± 9.2 mg/dL, respectively; *p* = 0.04) and serum magnesium (1.96 ± 0.27 and 1.60 ± 0.26 mg/dL, respectively; *p* = 0.005) levels were significantly increased in those taking MgCl_2_ compared with the controls. A total of 34 (29.4%) people improved their glucose status (50.8% and 7.0% in the magnesium and placebo groups, respectively; *p* < 0.0005) [67].

If magnesium supplementation affects insulin sensitivity in patients with diabetes mellitus, it may also improve insulin sensitivity in obese individuals who are at risk of type 2 diabetes mellitus. Therefore, effects of magnesium supplementation in overweight, normomagnesemic individuals who had insulin resistance, but not type 2 diabetes mellitus were examined. Individuals were randomly assigned to receive either magnesium aspartate hydrochloride supplementation (*n* = 27) or a placebo (*n* = 25) for 6 months. As trial endpoints, several indices of insulin sensitivity (e.g., plasma glucose, serum insulin) were determined. Magnesium supplementation resulted in a significant improvement of fasting blood glucose and some insulin sensitivity indices compared to placebo. The results provide evidence that magnesium supplementation improves insulin sensitivity even in normomagnesemic, overweight, non-diabetic subjects emphasizing the need for an early optimization of magnesium status to prevent insulin resistance and subsequently type 2 diabetes [80].

**Figure 2:**
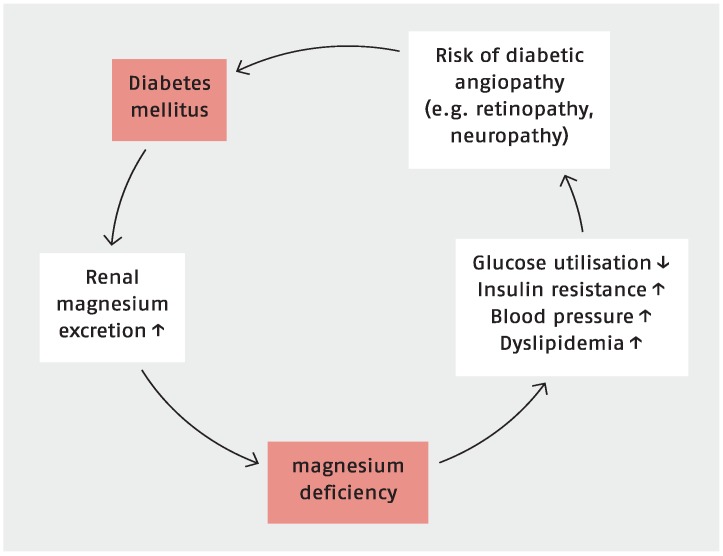
Magnesium deficiency and diabetes [63,71].

Diabetes is a disease that is strongly associated with both microvascular and macrovascular complications. Therefore, diabetes is a major public health problem associated with a huge economic burden in developing countries. These complications are wide ranging and are due at least in part to chronic elevation of blood glucose levels, which leads to damage of blood vessels. Among the most prevalent microvascular complications are kidney disease, blindness, and amputations. Impaired kidney function, exhibited as a reduced glomerular filtration rate, is also a major risk factor for macrovascular complications, such as heart attacks and strokes. Other chronic complications of diabetes include depression, dementia, and sexual dysfunction [18,72]. Magnesium depletion, for example by its effect on inositol transport, is of pathogenic significance in the development of diabetic complications (see Figure 2). A balanced magnesium status is associated with a decreased risk for microvascular and macrovascular complications [63,71,81,82,83,84]. Apart from this, magnesium intake or magnesium supplementation seems to have a positive impact in patients with diabetes or depression [85,86].

According to the recent guidelines of the Association for Magnesium Research, patients with diabetes benefit across four categories from magnesium supplementation: insulin sensitizing effect, calcium antagonism, stress regulating, and endothelium stabilizing effects. In diabetics, the Association for Magnesium Research recommends a daily magnesium supplementation between 240 and 480 mg (10–20 mmol) [17].

### 7.2. Cardiovascular Disease

#### 7.2.1. Hypertension

**Figure 3:**
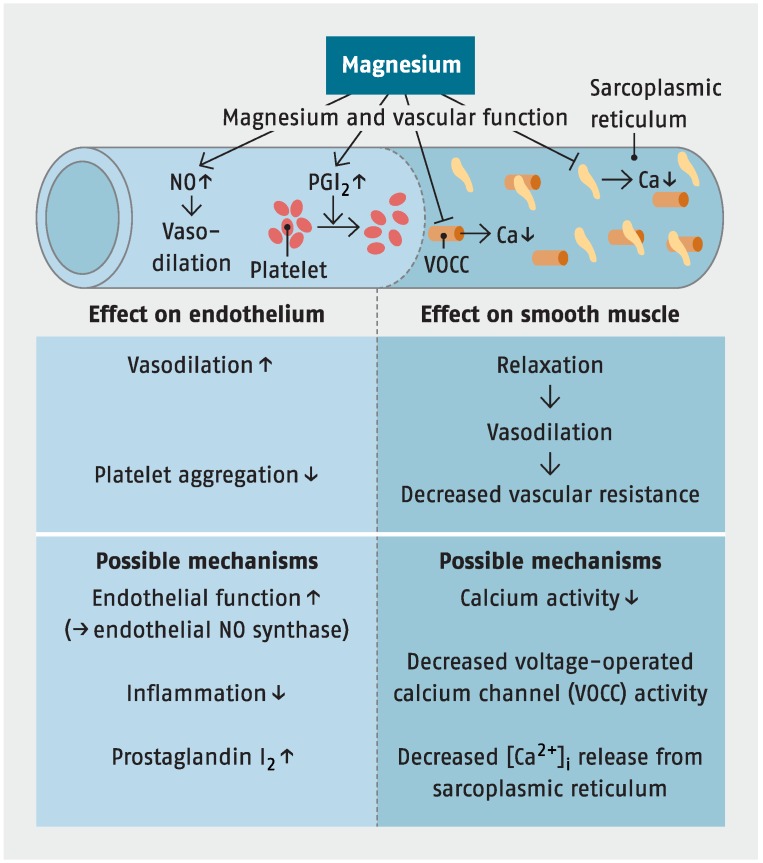
Magnesium and vascular function, according to [52,95].

A substantial body of epidemiological and experimental research is linking magnesium deficiency and cardiovascular diseases such as hypertension and atherosclerosis [22,71,87,88]. Hypertension is a major risk factor for heart disease and stroke. Magnesium is involved in blood pressure regulation. Every modification of the endogenous magnesium status leads to changes in vascular tonus and, as a consequence, changes in arterial blood pressure [71,89]. Magnesium deficiency increases angiotensin II-mediated aldosterone synthesis and the production of thromboxane and vasoconstrictor prostaglandins (see Figure 3) [47,70,74,95]. Furthermore, alterations in the metabolism of calcium and magnesium have been implicated in the pathogenesis of primary hypertension. Calcium influx across the external cellular membrane in smooth muscle cells and cardiomyocytes plays a crucial role in the control of cellular excitation contraction and impulse propagation. Intracellular calcium and magnesium concentrations are controlled by reversible binding to specific calcium-binding proteins. The calcium and magnesium flux across the external membrane is regulated by a calcium pump (calcium-magnesium-ATPase), calcium channels, and binding to the membrane. In cell membranes and in lymphocytes of hypertensive patients, our group showed significant increase of calcium, decrease of magnesium and an increased calcium/magnesium ratio (Ca^2+^/Mg^2+^ >2) [71,90,91,92,93,94]. In addition, it could be shown experimentally that a lack of magnesium increases the risk for lipid peroxidation and the development of dyslipoproteinemia.

In a meta-analysis of randomized trials, the effect of magnesium supplementation on blood pressure was tested. The 20 studies included 14 of hypertensive and six of normotensive persons totaling 1220 participants. The doses of magnesium ranged from 10–40 mmol per day (240–960 mg/day). The pooled net estimates of BP change (95% confidence interval (CI)) were −0.6 (−2.2 to 1.0) mm Hg for systolic blood pressure and −0.8 (−1.9 to 0.4) mm Hg for diastolic blood pressure. However, there was an apparent dose-dependent effect of magnesium, with reductions of 4.3 mm Hg systolic blood pressure (95% CI 6.3 to 2.2; *p* < 0.001) and of 2.3 mm Hg diastolic blood pressure (95% CI 4.9 to 0.0; *p* = 0.09) for each 10 mmol/day increase in magnesium dose [96]. Another meta-analysis of 12 randomized, controlled trials found that magnesium supplementation for 8–26 weeks in 545 hypertensive participants did not significantly reduce systolic blood pressure (mean difference: −1.3 mm Hg, 95% CI: −4.0 to 1.5, I(2) = 67%), but reduced significant diastolic blood pressure (mean difference: −2.2 mm Hg, 95% CI: −3.4 to −0.9, I(2) = 47%) [97]. A recent published meta-analysis of 22 trials with 1.173 normotensive and hypertensive adults concluded that magnesium supplementation for 3–24 weeks of follow up, decreased systolic blood pressure by 3–4 mm Hg and diastolic blood pressure by 2–3 mm Hg. The supplemental magnesium dose ranged from 120–973 mg/day. The effects were somewhat larger when supplemental magnesium intakes of the participants exceeded 370 mg/day [98]. A more recent meta-analysis, that examined 44 human studies involving oral magnesium supplementation for hypertension, and that were sorted according to hypertension status, magnesium dose and anti-hypertensive medication usage, found a significant lowering of blood pressure with magnesium supplementation while some studies reported no effect of magnesium. A uniform subset of seven studies from this meta-analysis involving 135 hypertensive subjects on anti-hypertensive medication continuously for at least six months, with no more than a two-week washout and with a mean starting systolic blood pressure (SBP) >155 mm Hg, demonstrated a mean change of −18.7 mm Hg (95% CI = −14.95 to −22.45, *p* < 0.0001) and an effect size test (Cohen’s d) = 1.19, *i.e.*, a large and highly significant effect. A meta-analysis of diastolic blood pressure (DBP) for these same seven studies showed a mean change in DBP of −10.9 mm Hg (95% CI = −8.73 to −13.1), *p* < 0.0001, with an effect size test (Cohen’s d) = 1.19 [32]. In borderline hypertension, decreased intracellular magnesium concentrations have recently been described. In patients with mild uncomplicated hypertension, respectively borderline hypertension, magnesium therapy can normalize blood pressure values [99,100]. Magnesium supplementation may also have a positive effect on resting and recovery systolic blood pressure with aerobic and resistance exercise [101]. Magnesium supplementation can help to control blood pressure and reduce the cardiovascular risk factors (e.g., atherosclerosis) associated with hypertension, especially in hypertensive individuals who are depleted of magnesium due to chronic diuretic use, inadequate intake, or both [22,32,96,98].

#### 7.2.2. Coronary Heart Disease, Myocardial Infarction and Stroke

Magnesium is a natural calcium antagonist and modulates vasomotor tone, blood pressure, and peripheral blood flow. Its actions as an antihypertensive, antidysrhythmic, anti-inflammatory and anticoagulant agent can be of benefit in the prevention and treatment of cardiovascular diseases. Recent experimental studies with Wistar rats reveal that short magnesium deficiency is associated with a downregulation of telomerase in left ventricular, right ventricular, atrial and aortic muscle cells. Furthermore a deficiency of magnesium resulted in these animal models in a 7–10 fold increased formation of 8-OH-dG in the cardiac and aortic muscle cells, and furthermore the magnesium deficiency is linked to an increased upregulation on neutral-sphingomyelinase (N-SMAse) and p53 in the cardiac and aortic muscle tissues [22]. Epidemiological studies have reported that serum and dietary magnesium are associated inversely with risk factors for coronary heart disease such as hypertension, type 2 diabetes mellitus, and the metabolic syndrome. Additional evidence from ecologic, clinical, and autopsy studies has shown higher magnesium to be potentially protective against sudden cardiac death. The Atherosclerosis Risk in Communities (ARIC) Study assessed risk factors and levels of serum magnesium in a cohort of 7887 women and 6345 men aged 45–64 years. After an average of 12 years of follow-up, individuals in the highest quartile of the normal physiologic range of serum magnesium (≥0.88 mmol/L) had an almost 40% reduced risk of sudden cardiac death compared with individuals in the lowest quartile (≤0.75 mmol/L) (HR: 0.62, 95% CI: 0.42–0.93) [102]. Another prospective study examined 88,375 women to determine whether serum magnesium levels measured early in the study and magnesium intakes from food and supplements assessed every 2–4 years were associated with sudden cardiac death over 26 years of follow-up. Women in the highest compared with the lowest quartile of daily ingested magnesium (<261 mg/day *vs.* >345 mg/day) and plasma magnesium concentrations (<0.78 mmol/L *vs.* >0.86 mmol/L) had a 37% (relative risk: 0.63; 95% CI: 0.44, 0.91) and 77% (relative risk: 0.23; 95% CI: 0.09, 0.60) lower risk of sudden cardiac death, respectively [29]. In the Prevention of Renal and Vascular End-Stage Disease (PREVEND) study, another prospective population-based cohort study with 7664 adults aged 20–75 years from The Netherlands found that low urinary magnesium excretion levels (a marker for low dietary magnesium intake) are associated with a higher risk of ischemic heart disease over a median follow-up period of 10.5 years [103]. The lowest sex-specific quintile (men: <2.93 mmol/24 h; women: <2.45 mmol/24 h) had an increased risk of fatal and nonfatal ischemic heart disease (multivariable HR: 1.60; 95% CI: 1.28, 2.00) compared with the upper four quintiles of urinary magnesium excretion [104].

A systematic review and meta-analysis of prospective studies that comprised 313,041 individuals and 11,995 cardiovascular diseases, 7534 ischemic heart diseases, and 2686 fatal ischemic heart disease events found that higher serum levels of magnesium were significantly associated with a lower risk of cardiovascular disease, and higher dietary magnesium intakes (up to approximately 250 mg/day) were associated with a significantly lower risk of ischemic heart disease caused by a reduced blood supply to the heart muscle. Circulating serum magnesium (per 0.2 mmol/L increment) was associated with a 30% lower risk of cardiovascular disease (RR: 0.70; 95% CI: 0.56, 0.88 per 0.2 mmol/L) and trends toward lower risks of IHD (RR: 0.83; 95% CI: 0.75, 1.05) and fatal ischemic heart disease (RR: 0.61; 95% CI: 0.37, 1.00). Dietary magnesium (per 200 mg/day increment) was not significantly associated with cardiovascular disease (RR: 0.89; 95% CI: 0.75, 1.05) but was associated with a 22% lower risk of ischemic heart disease (RR: 0.78; 95% CI: 0.67, 0.92). The association of dietary magnesium with fatal ischemic heart disease was nonlinear (*p* < 0.001), with an inverse association observed up to a threshold of ~250 mg/day (RR: 0.73; 95% CI: 0.62, 0.86), compared with lower intakes [105].

In a monocentric, controlled, double-blind study, 79 patients with severe congestive heart failure (NYHA IV) under optimal medical cardiovascular treatment were randomised to receive either magnesium orotate (6000 mg for 1 month, 3000 mg for about 11 months, *n* = 40) or placebo (*n* = 39). Both groups were comparable in demographic data, duration of heart failure and pre- and concomitant treatment. After mean treatment duration of 1 year (magnesium orotate: 364.1 +/− 14.7 days, placebo: 361.2 +/− 12.7 days) the survival rate was 75.7% compared to 51.6% under placebo (*p* < 0.05). Clinical symptoms improved in 38.5% of patients under magnesium orotate, whereas they deteriorated in 56.3% of patients under placebo (*p* < 0.001) [106]. In a recent study of our group with similar design we investigated hypertensives with heart insufficiency NYHA III-IV given additional magnesium therapy (magnesium orotate of about 2610 mg daily 3 times). The results showed in all magnesium treated hypertensive patients a positive effect on blood pressure, heart rhythm disorders and a lowering positive effect on NT-pro-BNP values as a marker for heart insufficiency. Pre-treatment NT-pro-BNP values decreased significantly in the magnesium orotate group already within 1 week (4761 +/− 2284 *versus* 3516 +/− 2114 pg/ml; *p* < 0.01, Wilcoxon-Test) [107,108]. Magnesium orotate may be used as adjuvant therapy in patients on optimal treatment for severe congestive heart failure, increasing survival rate and improving clinical symptoms and patient’s quality of life.

A meta-analysis of seven prospective trials with a total of 241,378 participants observed a modest but statistically significant inverse association between magnesium intake and risk of stroke. An intake increment of 100 mg Magnesium/day was associated with an 8% reduction in risk of total stroke (combined RR: 0.92; 95% CI: 0.88, 0.97). Magnesium intake was inversely associated with risk of ischemic stroke (RR: 0.91; 95% CI: 0.87, 0.96) but not intracerebral hemorrhage (RR: 0.96; 95% CI: 0.84, 1.10) or subarachnoid hemorrhage (RR: 1.01; 95% CI: 0.90, 1.14) [30]. In an updated meta-analyses of prospective studies to date, the combined RR of total stroke was 0.87 (95% CI: 0.83, 0.92) for a 100 mg/day increase in magnesium intake, 0.91 (95% CI: 0.88, 0.94) for a 1000 mg/day increase in potassium intake, and 0.98 (95% CI: 0.94, 1.02) for a 300 mg/day increase in calcium intake [109].

Magnesium sulfate is neuroprotective in preclinical models of stroke and has shown signals of potential efficacy with an acceptable safety profile when delivered early after stroke onset in humans. In a recent study, 1700 patients with suspected stroke received either intravenous magnesium sulfate or placebo, beginning within 2 h after symptom onset. Prehospital initiation of magnesium sulfate therapy was safe and allowed the start of therapy within 2 h after the onset of stroke symptoms, but it did not improve disability outcomes at 90 days [110].

In hemodialysis patients, low magnesium status is associated with other risk factors for cardiovascular disease such as greater incidence of intradialytic hypotension, poorer hemodialysis adequacy, deteriorating calcium-phosphate metabolism, inflammation and carotid intima-media thickness [111].

Cardiac arrhythmias are well known to be associated with hypomagnesaemia, although the contribution of hypomagnesaemia to its pathogenesis is not fully known due to coexisting hypokalaemia and other electrolyte disturbances. Possible effects of magnesium in preventing cardiac arrhythmias are stabilization of electrolyte concentrations of the heart muscle cell and membranes, calcium antagonism, elevation of cell energy niveau, improvement in O_2_ utilisation and diminishing of neurotransmitter release, e.g., adrenaline or noradrenaline. Magnesium depletion increases susceptibility to arrhythmogenic effects of drugs such as cardiac glycosides. The spectrum includes supraventricular and ventricular arrhythmias. Magnesium has a well-established role in the management of torsade de pointes. Torsade de pointes, a repetitive polymorphous ventricular tachycardia with prolongation of QT intervals, has been reported in cases of hypomagnesaemia, and this and other arrhythmias have been successfully treated with intravenous magnesium. In the recent guideline of the American Heart Association and the American College of Cardiology for prevention and treatment of torsade de pointes, tachycardia administration of magnesium and potassium is advised [10,26,40,59,112].

The frequency of cardiac arrhythmias occurring after myocardial infarction is higher in hypomagnesemic patients and can be reduced by magnesium administration. Several trials indicate that an intravenous magnesium infusion early after suspected myocardial infarction could decrease the risk of death. A meta-analysis with 2316 patients of the Leicester Intravenous Magnesium Intervention Trial (LIMIT-2) found a significant reduction in mortality in those patients who were given intravenous magnesium sulfate (8 mmol over 5 min followed by 65 mmol over 24 h) within 24 h of suspected myocardial infarction or physiological saline. By intention-to-treat analysis mortality from all causes was 7.8% in the magnesium group and 10.3% in the placebo group (2 *p* = 0.04), a relative reduction of 24% (95% confidence interval 1%–43%) [113]. However, another study involving 58,050 patients with suspected myocardial infarction, (ISIS-4, Fourth International Study of Infarct Survival), showed no benefit from magnesium therapy [114]. Also in the Magnesium in Coronaries (MAGIC) trial with 6213 patients with acute ST-elevation myocardial infarction, magnesium therapy had no benefit [114,115]. Thus, the use of intravenous magnesium sulphate remains controversial. Nevertheless, magnesium therapy should be considered in those with refractory arrhythmias.

### 7.3. Pre-Eclampsia and Eclampsia

Pre-eclampsia or preeclampsia is a disorder of pregnancy characterized by hypertension, proteinuria, often accompanied by pathological oedema. If left untreated, it may result in seizures at which point it is known as eclampsia. This complex disorder is characterized by haemoconcentration, vasoconstriction with increased peripheral resistance and reductions in cardiac output, plasma volume and prostacyclin synthesis. Up until today, magnesium sulfate has remained the most frequently used agent in the management of pre-eclampsia and eclampsia. Magnesium is the drug of choice to prevent convulsions in eclampsia [12,15,52,116]. In the Magpie trial, women (*n* = 5071) allocated magnesium sulfate had a 58% lower risk of eclampsia (95% CI 40–71) than those allocated placebo (*n* = 5070) [95]. The specific mechanisms of action remain are unclear, the effects of magnesium sulfate in the prevention of eclampsia are likely multi-factorial. Magnesium sulfate may act as a vasodilator, with actions in the peripheral vasculature or the cerebral vasculature, to decrease peripheral vascular resistance or relieve vasoconstriction. Additionally, magnesium sulfate may also protect the blood–brain barrier and limit cerebral edema formation, or it may act through a central anticonvulsant action [116]. Notably, nimodipine, a selective cerebral vasodilator, and also the antiepileptic phenytoine were not found to be as effective in eclampsia as magnesium [52,117].

### 7.4. Migraine Headaches

Studies have found that patients with cluster headaches and classic or common migraine, especially menstrual migraine, have low levels of magnesium [118,119,120]. In order to evaluate the prophylactic effect of oral magnesium, 81 patients aged 18–65 years with migraine according to the International Headache Society criteria (mean attack frequency 3.6 per month) were examined [121]. After a prospective baseline period of 4 weeks they received oral 600 mg (24 mmol) magnesium (trimagnesium dicitrate) daily for 12 weeks or placebo. In weeks 9–12 the attack frequency was reduced by 41.6% in the magnesium group and by 15.8% in the placebo group compared to the baseline (*p* < 0.05). The number of days with migraine and the drug consumption for symptomatic treatment per patient also decreased significantly in the magnesium group [122]. For acute treatment of migraine, intravenous magnesium sulfate (1000 mg magnesium intravenously) showed a statistically significant improvement in the treatment of all symptoms in patients with aura, or as an adjuvant therapy for associated symptoms in patients without aura [123]. According to recent studies, magnesium sulfate is as effective and a fast-acting medication compared to a combination of dexamethasone/metoclopramide for the treatment of acute migraine headaches [124].

### 7.5. ADHD

Attention deficit hyperactivity disorder (ADHD) is the most common psychiatric disorder in clinical samples of children and adolescents referring to child psychiatric clinics. Dietary factors can play a significant role in the etiology of attention deficit hyperactivity disorder (ADHD). Several studies reported that the magnesium level in children with ADHD is decreased in serum and erythrocytes and the Mg^2+^-ATPase activity is reduced [125]. Treatment of magnesium deficiency can help in revealing hyperactivity in children [126,127,128,129,130]. Current treatments for ADHD, such as atomoxetine and stimulants, act through adrenergic and dopaminergic receptors. Magnesium interacts with the ADHD-related neurotransmitters (e.g., dopamine, serotonin) and inhibits *N*-methyl-d-aspartate (NMDA)-induced norepinephrine release. The results of several studies are promising that magnesium supplementation (e.g. 6 mg/kg BW per day) may be helpful in the treatment of ADHD [126,127,128,129,130]. Unfortunately, until now there is still no double-blind randomized controlled clinical trial investigating the efficacy and safety of magnesium for treating ADHD.

### 7.6. Alzheimer’s Disease

Alzheimer’s disease (AD) is the most widespread reason for dementia. AD is characterized by profound synapse loss and impairments of learning and memory. Recent studies have demonstrated that the brain, serum and ionized magnesium levels are decreased in AD patients; however, the exact role of magnesium in AD pathogenesis remains unclear. In mice a chronic reduction in dietary magnesium impairs memory [131], and the treatment of dementia patients with nutritional magnesium improves memory [132,133]. Magnesium depletion, particularly in the hippocampus, appears to represent an important pathogenic factor in AD [134]. Magnesium affects many biochemical mechanisms that are vital for neuronal properties and synaptic plasticity. Magnesium treatment reduced Aβ plaque and prevented synapse loss and memory decline in a transgenic mouse model of AD [135]. A decreased magnesium level is found in various tissues of AD patients in clinical and laboratory studies [31,132]. New findings in animal studies are promising and provide novel insights in the neuroprotective effects of magnesium suggesting that magnesium treatment at the early stage may decrease the risk for cognitive decline in AD [136].

### 7.7. Asthma

Several clinical trials examined the effect of intravenous magnesium infusions on acute asthma attacks. A double-blind placebo-controlled trial in 38 adults who did not respond to initial treatment (beta agonist) in the emergency room found improved lung function and decreased likelihood of hospitalization when magnesium sulfate (1.2 g of magnesium sulfate) was infused compared with a placebo. Intravenous magnesium sulfate may represent a beneficial adjunct therapy in patients with moderate to severe asthma who show little improvement with beta-agonists [137]. In children with acute asthma, intravenous magnesium sulphate demonstrated also probable benefit in moderate to severe asthma in conjunction with standard bronchodilators and steroids [138,139]. A recent Cochrane review indicated that nebulised inhaled magnesium sulfate in addition to beta2-agonist in the treatment of an acute asthma exacerbation, appears to have also benefits with respect to improved pulmonary function in patients with severe asthma and there is a trend towards benefit in hospital admission [140].

### 7.8. Miscellaneous

Some of the potential indications that require further investigation include for example depression [141], dysmenorrhea [142], fatigue [143], fibromyalgia [144], hearing loss [145], kidney stones [146], premenstrual syndrome [147], osteoporosis [11], and tinnitus [148].

## 8. Dosage and Supplements

Many nutritional experts feel the ideal intake for magnesium should be based on the body weight (e.g., 4–6 mg per kg/day). Magnesium supplements are available as magnesium oxide, magnesium chloride, magnesium citrate, magnesium taurate, magnesium orotate, as well as other amino acid chelates. In the treatment of magnesium deficiency we would recommend, because of their high bioavailability, organic bound magnesium salts, such as magnesium citrate, gluconate, orotate, or aspartate [149].

## 9. Adverse Effects and Interactions

Magnesium supplementation is well tolerated, but it can cause gastrointestinal symptoms, including diarrhea, nausea, and vomiting. An overdose of intravenous magnesium may cause thirst, hypotension, drowsiness, muscle weakness, respiratory depression, cardiac arrhythmia, coma, and death. Concomitant use of magnesium and urinary excretion-reducing drugs, such as glucagons, calcitonin, and potassium-sparing diuretics, may increase serum magnesium levels, as may doxercalciferol. Concomitant oral intake of magnesium may influence the absorption of aminoglycosides, bisphosphonates, calcium channel blockers, fluoroquinolones, skeletal muscle relaxants and tetracylines. Therefore, concomitant use with these drugs should be avoided when possible. Attention and caution should be paid in patients with renal insufficiency (creatinine clearance: <30 mL per minute (0.50 mL per second)), because of the increased risk of heart block or hypermagnesemia [7].

## 10. Conclusions

Magnesium is an essential electrolyte for living organisms. Magnesium intoxication is rare. A magnesium deficiency is associated with a variety of diseases. In humans, a magnesium deficiency is associated with cardiovascular diseases, e.g., hypertension, pre-eclampsia, arrhythmias, heart failure. Arteriosclerosis, diabetes mellitus, and metabolic syndrome often occur in magnesium deficient humans. Furthermore, neurological symptoms are strengthened in magnesium deficient patients. Magnesium supplementation in those patients can be of benefit in most cases.
